# Temporal trends, in-hospital outcomes, and risk factors of acute myocardial infarction among patients with epilepsy in the United States: a retrospective national database analysis from 2008 to 2017

**DOI:** 10.3389/fneur.2024.1378682

**Published:** 2024-08-05

**Authors:** Zhemin Pan, Xi Xu, Shengyong Wu, Xi Chen, Xiao Luo, Chenxin Chen, Peimin Yu, Yingyi Qin, Jia He

**Affiliations:** ^1^Tongji University School of Medicine, Shanghai, China; ^2^Department of Urinary Surgery, Changhai Hospital, Naval Medical University, Shanghai, China; ^3^Department of Military Health Statistics, Navy Medical University, Shanghai, China; ^4^Department of Epidemiology and Statistics, School of Public Health, Medical College, Zhejiang University, Hangzhou, Zhejiang, China; ^5^Department of Neurology, Huashan Hospital, Fudan University, Shanghai, China

**Keywords:** acute myocardial infarction, atherosclerotic cardiovascular disease, clinical outcomes, dyslipidemia, epilepsy, prevalence, risk factors

## Abstract

**Background:**

The relationship between epilepsy and risk of acute myocardial infarction (AMI) is not fully understood. Evidence from the Stockholm Heart Study indicates that the risk of AMI is increased in people with epilepsy. This study aims to analyze the temporal trends in prevalence, adverse clinical outcomes, and risk factors of AMI in patients with epilepsy (PWE).

**Methods:**

Patients aged 18 years or older, diagnosed with epilepsy with or without AMI and hospitalized from January 1, 2008, to December 31, 2017, were identified from the National Inpatient Sample (NIS) database. The Cochran–Armitage trend test and logistic regressions were conducted using SAS 9.4. Odds ratios (ORs) were generated for multiple variables.

**Results:**

A total of 8,456,098 inpatients were eligible for our analysis, including 181,826 comorbid with AMI (2.15%). The prevalence of AMI diagnosis in PWE significantly increased from 1,911.7 per 100,000 hospitalizations in 2008 to 2,529.5 per 100,000 hospitalizations in 2017 (*P_trend_* < 0.001). Inpatient mortality was significantly higher in epilepsy patients with AMI compared to those without AMI (OR = 4.61, 95% CI: 4.54 to 4.69). Factors significantly associated with AMI in PWE included age (≥75 years old vs. 18 ~ 44 years old, OR = 3.54, 95% CI: 3.45 to 3.62), atherosclerosis (OR = 4.44, 95% CI: 4.40 to 4.49), conduction disorders (OR = 2.21, 95% CI: 2.17 to 2.26), cardiomyopathy (OR = 2.11, 95% CI: 2.08 to 2.15), coagulopathy (OR = 1.52, 95% CI: 1.49 to 1.54), dyslipidemia (OR = 1.26, 95% CI: 1.24 to 1.27), peptic ulcer disease (OR = 1.23, 95% CI: 1.13 to 1.33), chronic kidney disease (OR = 1.23, 95% CI: 1.22 to 1.25), smoking (OR = 1.20, 95% CI: 1.18 to 1.21), and weight loss (OR = 1.20, 95% CI: 1.18 to 1.22).

**Conclusion:**

The prevalence of AMI in PWE increased during the decade. Mortality rates were high among this population, highlighting the need for comprehensive attention to prophylaxis for risk factors and early diagnosis of AMI in PWE by physicians.

## 1 Introduction

Epilepsy, a complex disease causing a substantial burden on individuals and society, affects approximately 50 million people worldwide (1). Additionally, 2.4 million new cases are diagnosed each year (2). Despite its prevalence and severity, effective treatments remain limited. Throughout the course of “living with epilepsy,” patients may face various comorbidities, including acute myocardial infarction (AMI).

AMI is a leading cardiovascular disease, associated with high morbidity and mortality, resulting in approximately 9 million deaths annually (3). Recent researches suggest potential links between epilepsy and AMI, involving shared underlying pathophysiological mechanisms (4–6). Certain types of seizures are accompanied by cardiac complications, for instance, chronic epilepsy followed by seizure-induced cardiomyopathy causes abnormal remodeling of the heart, which may be leveraged by frequent hypoxia from spasms of the trachea and bronchus as well as their branches during seizures (7, 8). The anti-seizure medications, especially the enzyme-inducing anti-seizure medications (EIASMs) including carbamazepine, might substantially raise cholesterol levels, resulting in the increment of atherosclerosis and ensuing AMI (9). Furthermore, carbamazepine may increase the occurrence of AMI by inhibiting the electronic activity of myocardial cells when treating patients with epilepsy (10). Given these factors, patients with epilepsy (PWE) are at an increased risk for AMI. And, the co-occurrence of AMI in PWE might exacerbate the overall disease burden, extending beyond the challenges posed by seizures alone.

However, there are relatively few studies addressing this issue. Moreover, in real-world clinical settings, the adverse outcomes associated with the coexistence of epilepsy and AMI are often severely underestimated. Thus, in this study, we aimed to leverage the National (Nationwide) Inpatient Sample (NIS) database, the largest all-payer database in the United States, to explore the up-to-date situation and temporal trends of concomitant AMI in PWE, to compare the clinical outcome profiles between PWE with and without AMI, and to identify the independent risk factors of AMI, wishing to provide more precise and unearthed evidence for better management of the PWE.

## 2 Materials and methods

### 2.1 Data source and study population

Data from 2008 to 2017 in the NIS database were retrieved. The NIS is the largest all-payer database of hospital inpatient stays in the United States, containing discharge data from a 20% stratified sample of community hospitals. Using the sampling weights provided by the NIS, national estimates can be achieved (11). Since the NIS is a publicly available de-identified inpatient healthcare database, Institutional Review Board approval was not required according to the Healthcare Cost and Utilization Project (HCUP) data guidelines.

Using the NIS data from January 1, 2008, to December 31, 2017, a retrospective cohort study of inpatient PWE aged >18 years with or without subsequent AMI was conducted. The inclusion criteria were: (1) admitted from January 1, 2008, to December 31, 2017; and (2) had a diagnosis of epilepsy. Epilepsy in the diagnosis field was identified using International Classification of Diseases-9th Revision-Clinical Modification (ICD-9-CM) diagnostic or International Classification of Diseases-10th Revision-Clinical Modification (ICD-10-CM) diagnostic codes (12). AMI was defined in cases where the diagnosis included AMI, identified by ICD-9-CM or ICD-10-CM codes (Supplementary Table A1) (13). Records that met the following criteria were excluded: (1) age < 18 years old; (2) length of hospital stay ≤1 day; (3) total cost = 0; (4) missing in-hospital death status; and (5) transferred to another hospital or institution on the day of admission.

### 2.2 Primary and secondary outcomes

The primary outcome of this study was temporal trends in the prevalence of AMI in PWE. Secondary outcomes included rates and trends of the inpatient mortality among PWE with or without AMI; associations of AMI with inpatient adverse clinical outcomes, cost and length of stay (LOS); independent risk factors associated with AMI in PWE; and trends of these factors.

### 2.3 Statistical analysis

Descriptive statistics were used to summarize the characteristics and demographics of all hospitalizations. Univariate analysis was performed for baseline characteristics and the outcomes between two groups: the PWE with AMI group and the PWE without AMI group. For continuous variables, the Kolmogorov–Smirnov test was first employed to evaluate the normality of the distribution (14). The independent *t*-test was then used for normally distributed continuous variables, and the Wilcoxon rank-sum test was used for non-normally distributed continuous variables. For categorical variables, the Chi-square test was employed. The Cochran–Armitage trend test was used to evaluate the significance of trends over time (15, 16). To assess the association between AMI and adverse inpatient clinical outcomes, cost, and LOS, univariate logistic regression models (unadjusted model 1) and multivariate-adjusted logistic regression models (17) (model 2 and 3) were implemented. Both model 2 and model 3 were adjusted for patient-level and hospital-level characteristics, and comorbidities (Supplementary Table A2). Missing variables were imputed in model 2 as the main analysis, while the original unimputed data were used in model 3 as the sensitivity analysis. Missing values in a categorical variable were imputed by the corresponding dominant category, and missing values in a continuous variable were imputed by the corresponding median or mean value, depending on the results of the normality test (18). Using the same covariates and missing data imputation method, univariate and multivariate-adjusted logistic regression models were also applied to explore the potential risk factors associated with AMI and epilepsy. Subgroup analyses were also performed based on the subtypes of AMI, including non-ST-segment elevation myocardial infarction (NSTEMI) and ST-segment elevation myocardial infarction (STEMI). According to the HCUP guidance, sampling weights were applied to generate the national estimates (19, 20). Every possible comparison between the study groups was considered. All tests were two-tailed with a significant level set at *p* ≤ 0.05. All analyses were conducted utilizing SAS 9.4 (SAS Institute Inc. Cary, NC).

## 3 Results

### 3.1 Study population

Among the 8,456,098 patients aged ≥18 years who were diagnosed with epilepsy from 2008 to 2017 and included in the study, 181,826 (2.15%) had concomitant AMI. Baseline characteristics are summarized in Supplementary Table A3. In the dataset analyzed, the majority of variables exhibited a missing rate of less than 2% among all hospitalizations. Race and median household income displayed higher missing rates of 6.89 and 2.74%, respectively.

### 3.2 Prevalence and temporal trends of AMI in PWE

**Figure 1:**
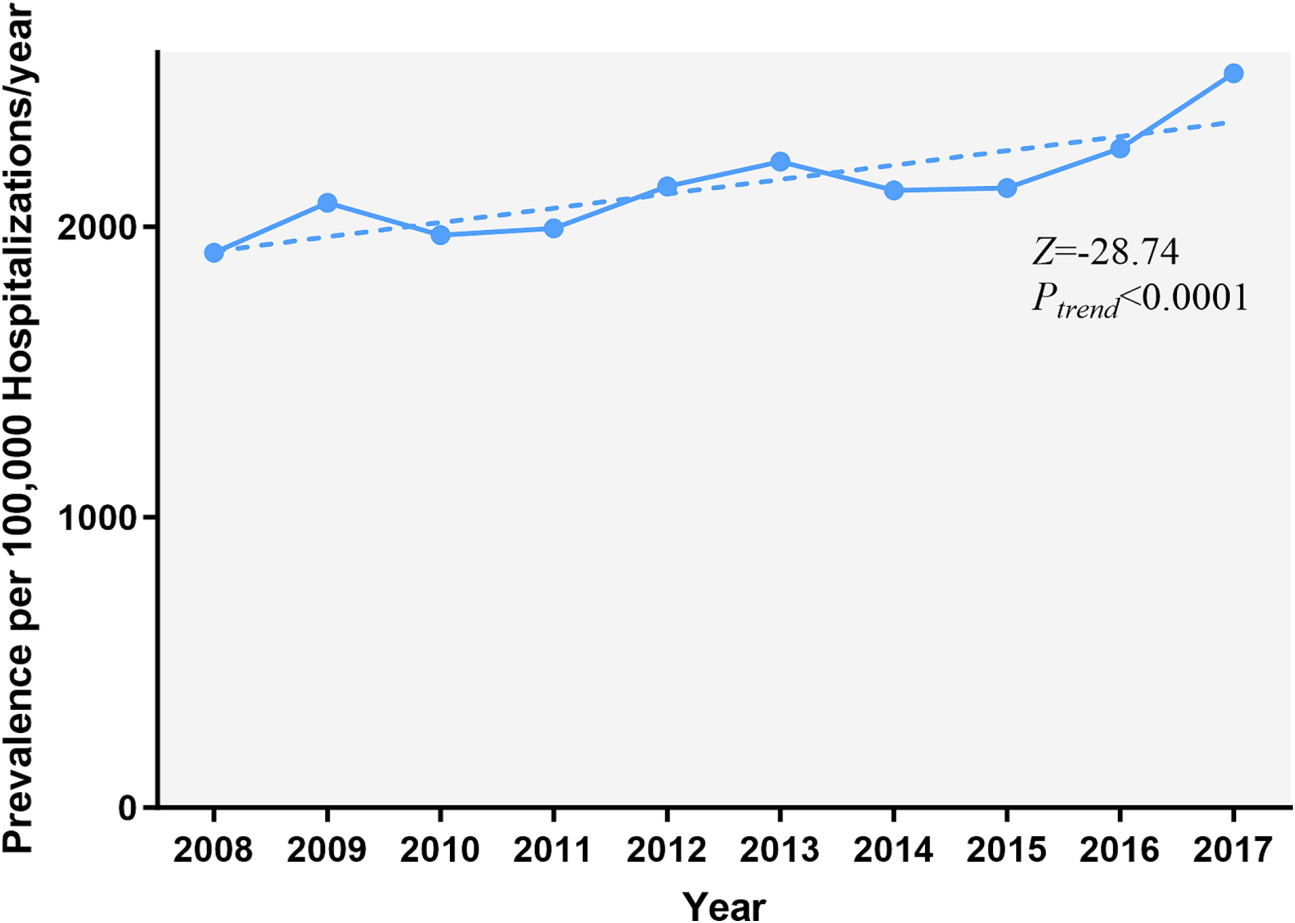
Temporal trends of AMI in PWE. The prevalence of AMI in PWE increased significantly from 2008 to 2017 (*P_trend_* < 0.001). The significance of trends over time was assessed using the Cochran-Armitage trend test. AMI, acute myocardial infarction; PWE, patients with epilepsy.

**Figure 2:**
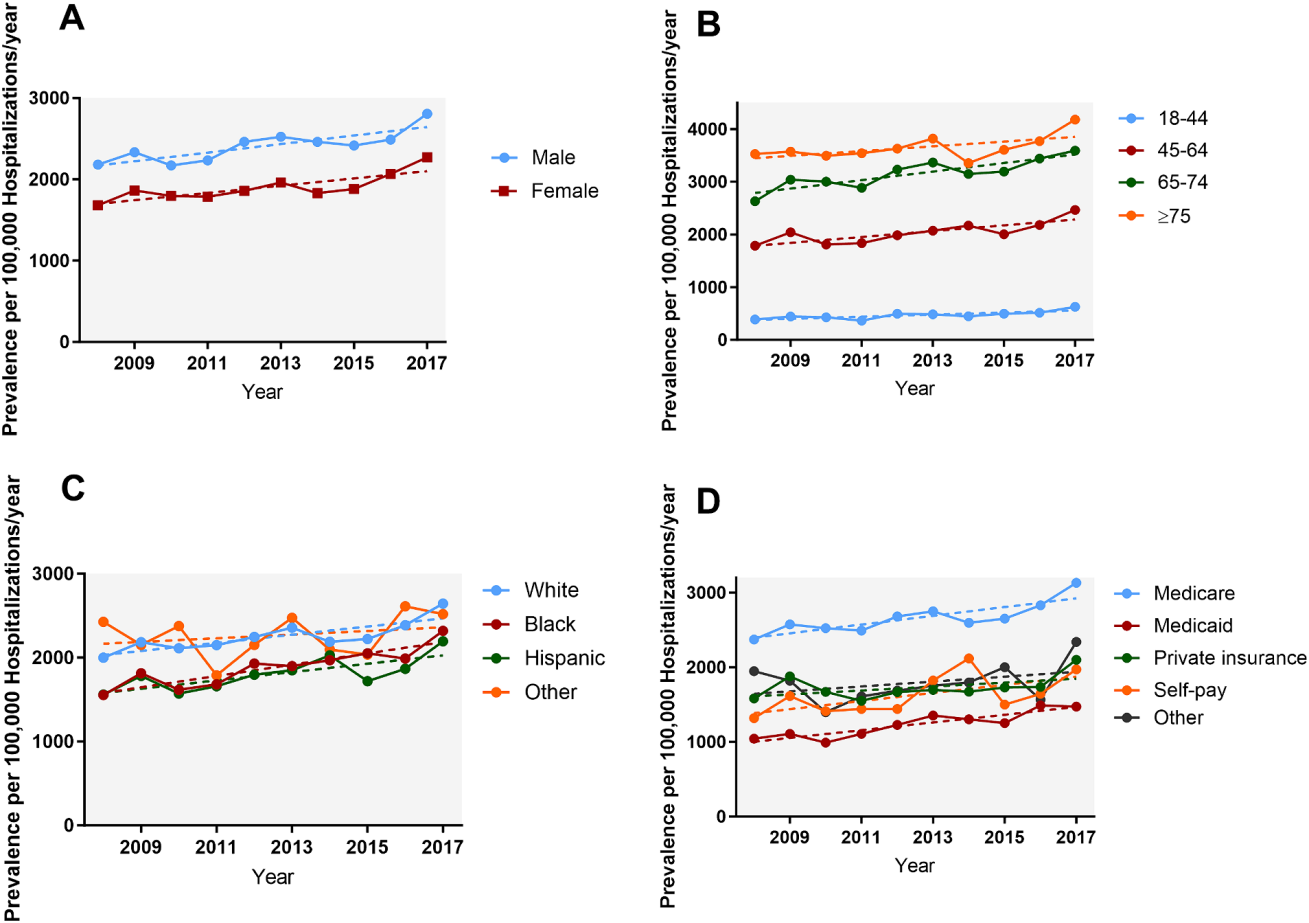
Temporal trends of AMI in PWE subgroups categorized by sex **(A)**, age **(B)**, race **(C)**, and insurance type **(D)**. From 2008 to 2017 the prevalence of AMI in PWE increased across all subgroups (all *P_trend_* < 0.05). AMI, acute myocardial infarction; PWE, patients with epilepsy.

The prevalence of AMI increased over 10 years, from 1,911.7 cases per 100,000 epilepsy hospitalizations in 2008 to 2,529.5 in 2017 (*P_trend_* < 0.001) (Figure 1). Figure 2 and Supplementary Table A4 show the temporal trends of AMI prevalence in subgroups categorized by sex, age, race, household income, insurance type, and hospital region. From 2008 to 2017, AMI prevalence in PWE increased across all subgroups. It increased with age, being 6–9 times higher in the ≥75 years old group than in the 18–44 years old group, and it showed higher prevalence in men than in women.

Supplementary Figure A1 illustrates the increasing trend in both STEMI and NSTEMI cases over the past decade (*P_trend_* < 0.001). The trend of NSTEMI surges more conspicuously from visual inspection.

### 3.3 Association between AMI and adverse in-patient clinical outcomes

The in-patient mortality rate was higher in PWE with AMI (13.35%) compared to those without AMI (2.5%). Similarly, AMI was associated with a significantly higher risk of acute heart failure, acute respiratory failure, acute renal failure, cardiogenic shock, venous thromboembolism, fluid and electrolyte disorders, and neurological failure, as well as higher hospital costs and longer LOS (Supplementary Table A5).

**Figure 3:**
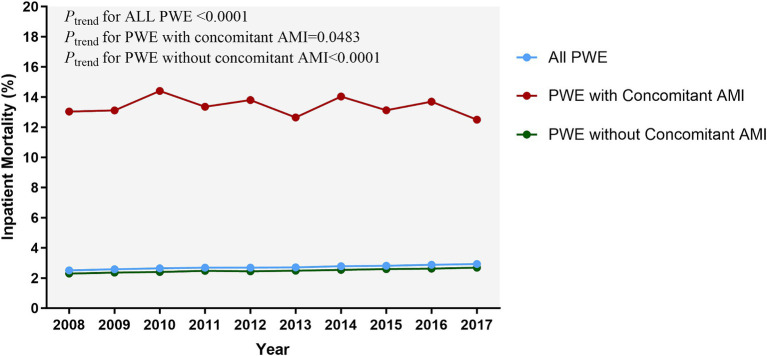
Temporal trends of inpatient mortality related to epilepsy. The inpatient mortality in PWE with AMI mildly decreased but remained high during the study period (13.04% in 2008 vs. 12.50% in 2017); (*P_trend_* = 0.0483). The significance of trends over time was assessed using the Cochran-Armitage trend test. AMI, acute myocardial infarction; PWE, patients with epilepsy.

The trends of in-hospital mortality remained relatively stable in both groups, with a modest decrease observed among patients with AMI and a subtle increase among those without AMI (Figure 3).

**Figure 4:**
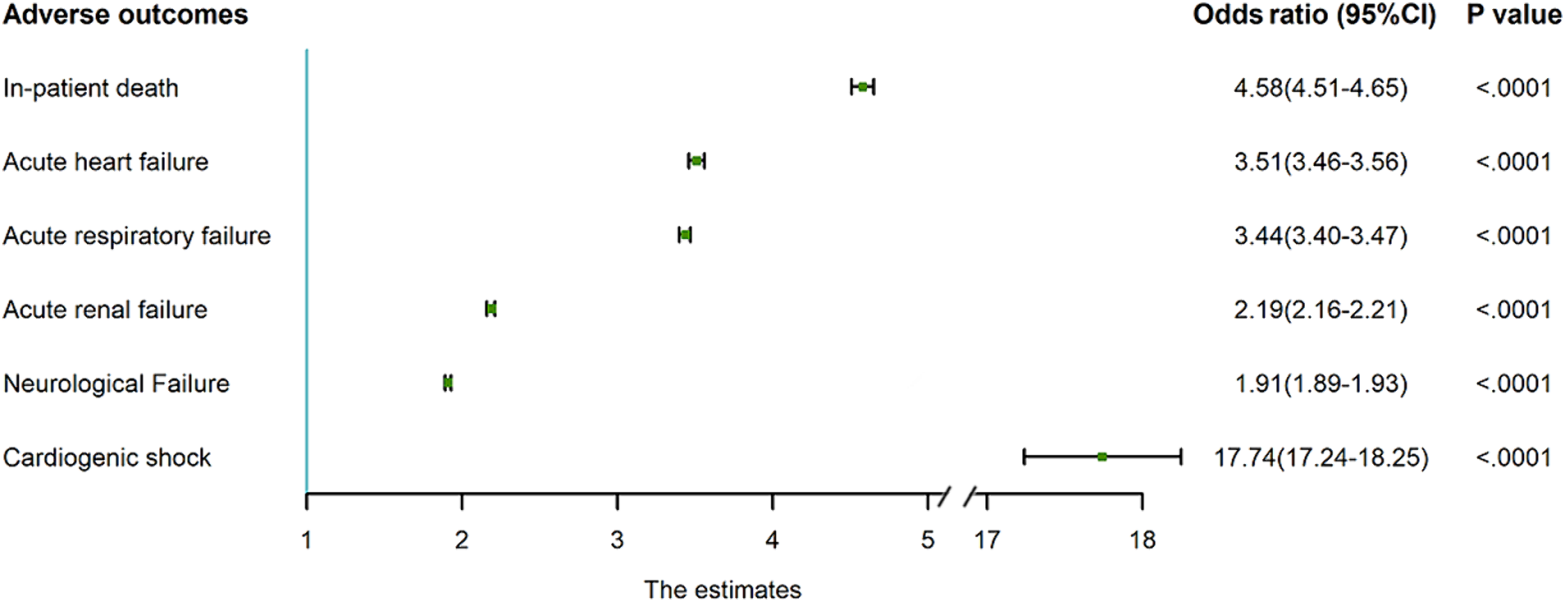
Association of concomitant AMI with adverse clinical outcomes in PWE. Concomitant AMI was independently associated with higher risk of in-patient death, acute heart failure, acute respiratory failure, acute renal failure, neurological failure, and cardiogenic shock. Obtained from multivariate logistic regression models with each adverse outcome as the dependent variable and concomitant AMI as the independent variable, adjusted for patient-level factors (sex, age, race, insurance type, income level, discharge year and season), hospital-level factors (hospital ownership, hospital region, hospital size, hospital location, teaching status), and comorbidity related factors. The analysis accounted for the survey design and was based on the imputed dataset (missing values in category variables were imputed by the dominant category, and missing values in continuous variables were imputed by the median value). AMI, acute myocardial infarction; PWE, patients with epilepsy; CI, confidence interval.

In the multivariable logistic regression analysis, which adjusted patient-level, hospital-level and comorbidity related factors, AMI was independently associated with increased inpatient death (OR = 4.61, 95% CI: 4.54 to 4.68), acute heart failure (OR = 3.51, 95% CI: 3.46 to 4.56), acute respiratory failure (OR = 3.44, 95% CI: 3.40 to 3.47), acute renal failure (OR = 2.19, 95% CI: 2.16 to 2.21), neurological failure (OR = 1.91, 95% CI: 1.89 to 1.93), and heart attack (OR = 17.74, 95% CI: 17.24 to 18.25) (Figure 4; Supplementary Table A5). AMI was also related to an increased cost of $44,567 and an increased LOS of 1.685 days after comprehensively adjusting for confounders (Table 1). The robustness of these results was confirmed by the sensitivity analysis (Supplementary Table A6).

**Table 1:** Association of concomitant AMI with hospitalization cost and LOS in PWE.

Items	Estimates	95% CI	*p*-value
**Association of AMI compared with no AMI with each outcome among all participants**			
Cost	$44,567	$43,655 to $45,480	<0.001
LOS, days	1.69	1.60 to 1.77	<0.001
**Association of each outcome with unit increase in year when restricted to participants with comorbid AMI**			
Cost	$2,959.59	$2,439.75 to $3,479.43	<0.001
LOS, days	−0.11	−0.15 to −0.08	<0.001
**Association of each outcome with unit increase in year when restricted to participants without comorbid AMI**			
Cost	$1,665.21	$1,617.47 to $1,712.95	<0.001
LOS, days	−0.04	−0.05 to −0.04	<0.001

Obtained from multivariate linear regression model with either cost or LOS as the dependent variable and concomitant AMI as the independent variable, adjusted for patient-level factors (sex, age, race, insurance type, income level, discharge year, and season), hospital-level factors (hospital ownership, hospital region, hospital size, hospital location, teaching status), and comorbidity related factors. The analysis accounted for the survey design and was based on the imputed dataset (missing values in category variables were imputed by the dominant category, and missing values in continuous variables were imputed by the median value). AMI, acute myocardial infarction; LOS, length of stay; PWE, patients with epilepsy; CI, confidence interval.

Compared with PWE alone, the presence of either STEMI or NSTEMI in PWE significantly elevated the risk of adverse inpatient outcomes. Numerically, the risk of adverse clinical outcome seems higher in PWE with concomitant STEMI than in PWE with NSTEMI, especially in in-patient death and cardiogenic shock (Supplementary Table A8).

### 3.4 Risk factors of AMI in PWE

The following factors were found to be associated with concomitant AMI in PWE after adjusting for confounding variables: age ≥ 75 years (OR = 3.54, 95% CI: 3.45 to 3.62), no insurance (OR = 1.41, 95% CI: 1.37 to 1.45), admission on the weekend (OR = 1.21, 95% CI: 1.20 to 1.23), atherosclerosis (OR = 4.44, 95% CI: 4.40 to 4.49), conduction disorders (OR = 2.21, 95% CI: 2.17 to 2.26), cardiomyopathy (OR = 2.11, 95% CI: 2.08 to 2.15), coagulopathy (OR = 1.52, 95% CI: 1.49 to 1.54), dyslipidemia (OR = 1.26, 95% CI: 1.24 to 1.27), peptic ulcer disease excluding bleeding (OR = 1.23, 95% CI: 1.13 to 1.33), chronic kidney disease (CKD) (OR = 1.23, 95% CI: 1.22 to 1.25), smoking history (OR = 1.20, 95% CI: 1.18 to 1.21), and weight loss (OR = 1.20, 95% CI: 1.18 to 1.22) (Supplementary Table A7).

Trends in risk factor profiles are shown in Table 2. From 2008 to 2017, the prevalence of most risk factors increased, except for conduction disorders, chronic blood loss anemia, and uncomplicated diabetes.

**Table 2:** Temporal trends of the prevalence of risk factors for concomitant AMI in PWE.

Risk factors	OR (95% CI)	Prevalence (%)										*Z* value	*P_trend_*
		2008	2009	2010	2011	2012	2013	2014	2015	2016	2017		
Atherosclerosis	4.44 (4.40–4.49)	16.26	17.69	17.58	19.28	18.95	18.96	19.15	18.61	18.25	18.03	−25.99	<0.001
Conduction disorders	2.21 (2.17–2.26)	1.7	2.03	1.98	2.14	2.09	2.06	2.18	2.33	2.78	2.96	−63.43	<0.001
Cardiomyopathy	2.11 (2.08–2.15)	2.42	2.6	2.53	2.66	2.67	2.81	2.91	2.92	2.97	2.96	−31.27	<0.001
Coagulopathy	1.52 (1.49–1.54)	4.17	5.18	5.97	6.44	6.88	7.06	7.18	7.39	8.12	8.3	−130.34	<0.001
Dyslipidemia	1.26 (1.24–1.27)	19.29	21.54	23.37	25.84	27.49	28.34	29.32	30.35	30.88	32.01	−248.18	<0.001
Peptic ulcer disease	1.23 (1.13–1.33)	0.03	0.04	0.03	0.03	0.04	0.03	0.03	0.28	0.98	0.91	−163.35	<0.001
Chronic kidney disease	1.23 (1.22–1.25)	10.29	12.36	12.81	14.21	14.16	14.63	15.36	16.02	16.5	17.15	−153.55	<0.001
History of tobacco use	1.20 (1.18–1.21)	18.98	21.96	23.56	24.44	26.62	27.99	31.17	30.58	21.97	22	−78.21	<0.001
Weight loss	1.20 (1.18–1.22)	4.56	5.65	6.02	7.32	7.07	7.17	7.47	7.73	8.07	8.86	−122.62	<0.001
Drug abuse	1.18 (1.16–1.21)	5.99	6.65	6.83	7.04	7.67	8.3	8.84	8.98	7.48	7.59	−64.96	<0.001
Diabetes with chronic complication	1.15 (1.13–1.16)	3.66	3.99	4	4.58	4.64	4.89	5.34	6.71	11.59	15.29	−366.56	<0.001
Atrial fibrillation	1.12 (1.11–1.13)	8.12	9.17	9.35	10.43	10.78	11.14	11.64	12.25	12.71	13.33	−142.62	<0.001
Chronic blood loss anemia	1.10 (1.05–1.14)	1.29	1.31	1.26	1.21	1.2	1.14	1.16	1.2	1.22	1.18	9.51	<0.001
Obesity	1.09 (1.07–1.11)	6.83	7.85	7.82	9.16	9.88	10.59	11.19	11.75	12.19	12.98	−184.55	<0.001
Peripheral vascular disease	1.09 (1.08–1.11)	4.45	5.01	4.88	5.62	5.49	5.59	5.72	5.93	5.73	5.14	−33.69	<0.001
Diabetes without complication	1.08 (1.06–1.09)	17.51	18.56	18.81	19.74	19.87	19.77	19.57	18.53	13.77	10.93	140.16	<0.001
Deficiency anemias	1.08 (1.06–1.09)	18.67	20.47	20.81	22.92	22.59	22.43	22.37	22.58	22.68	22.57	−63.87	<0.001

ORs were obtained from a multivariate logistic regression model, with AMI as the dependent variable and comorbidity related factors as independent variables, adjusted for patient-level factors (sex, age, race, insurance type, income level, discharge year, and season), and hospital-level factors (hospital ownership, hospital region, hospital size, hospital location, and teaching status). The analysis accounted for the survey design and was based on the imputed dataset (missing values in category variables were imputed by the dominant category, and missing values in continuous variables were imputed by the median value). The significance of trends over time was assessed using the Cochran-Armitage trend test. OR, odds ratio; AMI, acute myocardial infarction; PWE, patients with epilepsy.

## 4 Discussion

In this study, by exploring a nationwide inpatient sample database, we found that AMI occurred in approximately 2.15% of hospitalizations involving PWE. Notably, the prevalence of AMI diagnosis in PWE significantly increased over time. Furthermore, AMI was strongly associated with increased inpatient mortality in PWE in both the adjusted and unadjusted models. Atherosclerosis, older age, conduction disorders, cardiomyopathy, coagulopathy, dyslipidemia, peptic ulcer disease, smoking, and weight loss were identified as independent risk factors for AMI in PWE. Nearly all of these risk factors showed an increasing trend from 2008 to 2017.

In this study, we found the average prevalence of AMI in PWE was approximately 2,150 per 100,000 hospitalizations per year, aligning with a recent retrospective cohort study conducted in South Carolina (6). However, our study’s AMI prevalence appeared to be ten times higher than that observed in general hospitalizations, which ranged from 2.4–3.1 per 1,000 hospitalizations per year (21). Nevertheless, recent studies have consistently shown a higher risk of AMI in PWE compared to individuals without epilepsy (5, 9), and our findings support this observation.

Given the widespread awareness of traditional cardiovascular disease risks and subsequent innovations in medication and risk factor management, the prevalence of heart attacks in the United States has appreciably decreased over the past two decades (22, 23). However, in contrast to the decreasing trends of AMI prevalence in the general public, this nationwide real-world study observed a significantly rising trend of AMI in PWE by almost 32% from 2008 to 2017. Notably, this trend was consistent across all age groups including young populations, although the proportion remained highest in patients over 75 years of age.

Several factors may explain the higher prevalence and rising trends of AMI in PWE. First, the increasing trends in almost all AMI risk factors in PWE likely contributed to the elevated prevalence of this condition. Second, physicians may overlook somatic comorbidities in PWE, contributing to higher AMI prevalence. Indeed, previous reports indicate that somatic comorbidities in PWE are significant but frequently neglected burdens (24, 25). While some of these somatic comorbidities are traditional risk factors for AMI (26), in clinical practice, doctors are often focused on controlling seizures, potentially neglecting the comprehensive management of epilepsy and its comorbidities, including AMI (27). Third, compliance issues might also lead to rising AMI prevalence, as evidence shows that PWE often struggle with understanding of their illness, complex drug regimens, costs, and adverse events caused by medications (28).

Given the rising trend of AMI in PWE, as well as the 4.61-fold higher mortality risk and 17.74-fold higher heart attack risk in this cohort, recognizing AMI risk factors in PWE and implementing effective prophylaxis is crucial.

Among all the AMI-related risk factors with an elevated trend, dyslipidemia was the most prominent, increasing significantly from 19.29% in 2008 to 32.01% in 2017. High levels of low-density lipoprotein (LDL) and low levels of high-density lipoprotein (HDL) are strongly associated with the onset of AMI (29, 30). Additionally, other risk factors appear to be directly and indirectly related to dyslipidemia. Atherosclerosis, for example, acts as a link between hyperlipidemia and AMI. Hyperlipidemia leads to lipid deposition beneath the arterial endothelium, forming macroscopic plaques. The rupture of these plaques is a common trigger of acute coronary artery thrombosis, leading to AMI (31).

Furthermore, peripheral arterial disease and AMI share common mechanisms. Dyslipidemia aggravates atherosclerosis, leading to plaque enlargement and subsequent peripheral vascular disease and coronary artery stenosis (32). Consequently, patients with peripheral vascular disease have high rates of simultaneous coronary atherosclerosis, resulting in a high risk of myocardial infarction in this population (33).

In addition to the fact that dyslipidemia is prevalent in patients with CKD (34), recent evidence illustrates its contribution to the pathogenesis of renal disease caused by impaired cholesterol efflux, which is known as the “lipid nephrotoxicity hypothesis” (35, 36). When CKD occurs, the kidney secretes hormones, enzymes, and cytokines in response, causing characteristic vascular changes. CKD-related mediators and hemodynamic alterations can also cause cardiac damage, accelerating cardiovascular disease progression, including AMI (37). Furthermore, abnormal glucose metabolism can lead to dyslipidemia, and lipid changes can result in type 2 diabetes (38). Elevated triglycerides lead to increased levels of free fatty acids, inducing insulin resistance and β-cell dysfunction (39, 40). A recent study also highlighted that higher HDL-C concentrations are negatively associated with hyperglycemia (41), and it is well established that patients with diabetes are an increased risk of AMI (42).

There is also a strong association between dyslipidemia and obesity. Dyslipidemias are common in obese patients (43), and insufficient fat oxidation can increase weight gain risk (44). Moreover, obese patients are at high risk for AMI, making lipid-lowering interventions essential.

Given the clear relevance of dyslipidemia and other AMI risk factors we identified, controlling serum lipid levels is crucial in preventing AMI in PWE (42). The propagation of lipid-modifying therapy and increased awareness of dyslipidemia among the public and physicians has led to a decline in serum lipid levels in the United States (22, 45–47). As mentioned earlier, this decline coincides with the decreased prevalence of AMI in the general population. However, PWE in our study had a high prevalence of dyslipidemia, consistent with previous literature (48, 49).

Three possible reasons might explain the high prevalence of dyslipidemia in PWE. First, the lifestyles of PWE could be a contributing factor (50). Second, the administration of EIASMs, such as carbamazepine, phenytoin and phenobarbital, could play a role. EIASMs are metabolized by hepatic cytochrome P450 enzymes, which competitively interfere with cholesterol catabolism to bile acids, catalyzed by the same enzyme system (51–54). Additionally, these medications are associated with mild hypothyroidism, potentially triggering hypercholesterolemia (55). Third, a lack of awareness of lipid management criteria in PWE may also play a role.

Our study highlights the significant disease burden of PWE with AMI: increasing prevalence, worse clinical outcomes, and high in-hospital mortality. Nevertheless, this study only included inpatient patients, and many PWE might experience “silent” myocardial infarctions without hospitalization. Therefore, attention is needed concerning the relationship between epilepsy and AMI. As such, when PWE experience chest pain, AMI should be immediately considered. Additionally, the threat of hyperlipidemia in PWE should be emphasized among physicians, with sufficient evaluation of ASM treatment considering of both seizure control and lipid management. Early diagnosis and prevention for PWE at higher risk of AMI are crucial. Moreover, accessible patient education, regular lipid monitoring and electrocardiograph are urgently needed. Finally, as widely acknowledged in the society, the vital risk factors for seizures in PWE are self-discontinue treatment, significant alcohol or illicit drug use, etc. (56). Thereby, controlling those risk factors may decrease the frequency of seizures, protecting the myocardial cells from hypoxia caused by spasm of respiratory muscles during seizures.

### 4.1 Limitations

The current analysis has some limitations. First, due to the population characteristics of NIS, we only investigated the condition in adult PWE. Thus, pediatric patients, who are more vulnerable to heart attack owing to the ketogenic diet followed by them, remain to be further studied. Second, the NIS database does not document some vital patient information, such as medications, etiologies, course, and genetic profiles. This limitation caused potential bias in evaluating outcomes and may omit possible risk factors. Third, since the NIS database is primarily retrospective, exploring the causal relationship between any two factors is challenging. Fourth, previous studies have elucidated a higher prevalence of AMI in patients with refectory epilepsy. However, the ICD code for refractory epilepsy is seldomly implemented, resulting in less classification of epilepsy in this database. This limitation advances the paucity of investigating the correlation between the two in this study.

## 5 Conclusion

The trend of concomitant AMI in PWE has been increasing over the past decade. Concomitant AMI could worsen outcomes and increase the risk of mortality in this population. More attention with regard to cardiovascular risks, especially AMI, should be addressed in PWE besides seizure control.

## Data availability statement

Publicly available datasets were analyzed in this study. This data can be found at: https://hcup-us.ahrq.gov/.

## Ethics statement

Ethical approval was not required for the study involving humans in accordance with the local legislation and institutional requirements. Written informed consent to participate in this study was not required from the participants or the participants' legal guardians/next of kin in accordance with the national legislation and the institutional requirements.

## Author contributions

ZP: Writing – original draft, Writing – review & editing. XX: Conceptualization, Investigation, Writing – original draft. SW: Formal analysis, Methodology, Writing – original draft. XC: Conceptualization, Investigation, Methodology, Validation, Writing – original draft. XL: Data curation, Validation, Visualization, Writing – original draft. CC: Data curation, Resources, Writing – original draft. PY: Supervision, Validation, Visualization, Writing – review & editing. YQ: Supervision, Writing – review & editing. JH: Supervision, Writing – review & editing.
