# Supplementary material for: Temporal trends, in-hospital outcomes, and risk factors of acute myocardial infarction among patients with epilepsy in the United States: a retrospective national database analysis from 2008 to 2017
Source: Front Neurol. 2024 Aug 5;15:1378682. doi: 10.3389/fneur.2024.1378682 (PMC11330761; doi:10.3389/fneur.2024.1378682)
Supplement: Supplementary file 1 [file Data_Sheet_1.docx]

## Table A.1. The ICD Codes Utilized in the Study

| Disease | ICD-9-CM | ICD-10-CM |
| --- | --- | --- |
| Acute Myocardial Infarction | 410.0, 410.1, 410.2, 410.3, 410.4, 410.5, 410.6, 410.7, 410.8, 410.9 | I21.0, I21.1, I21.2, I21.3, I21.4, I21.9, I22.0, I22.1, I22.8, I22.9 |
| Epilepsy | 345.00, 345.01, 345.10, 345.11, 345.2, 345.3, 345.40, 345.41, 345.50, 345.51, 345.60, 345.61, 345.70, 345.71, 345.80, 345.81, 345.90, 345.91 | G40.A01, G40.A09, G40.A11, G40.A19, G40.309, G40.401, G40.409, G40.311, G40.411, G40.419, G40.A01, G40.A09, G40.A11, G40.A19, G40.301, G40.201, G40.209, G40.211, G40.219, G40.101, G40.109, G40.111, G40.119, G40.821, G40.822, G40.823, G40.824, G40.101, G40.109, G40.111, G40.119, G40.101, G40.109, G40.501, G40.509, G40.802, G40.111, G40.119, G40.804, G40.901, G40.909, G40.911, G40.919 |

ICD-9-CM = International Classification of Diseases-9th Revision-Clinical Modification; ICD-10-CM = International Classification of Diseases-10th Revision-Clinical Modification

## Table A.2. Covariates Adjusted for in Multivariable Analysis in the Study

| Category | Covariates |
| --- | --- |
| Patient-level | age, sex, race, admission year, season, insurance type, income level |
| Hospital-level | control/ownership of hospital, hospital size (bed number), teaching status, location status of hospital, region of hospital |
| Comorbidity-related | acquired immune deficiency syndrome, alcohol abuse, deficiency anemias, rheumatoid arthritis/collagen vascular diseases, chronic blood loss anemia, chronic pulmonary disease, coagulopathy, depression, diabetes uncomplicated, diabetes with chronic complications, drug abuse, hypertension, hypothyroidism, liver disease, lymphoma, metastatic cancer, obesity, paralysis, peripheral vascular disorders, psychoses, solid tumor without metastasis, peptic ulcer disease excluding bleeding, valvular disease, weight loss, refectory epilepsy, dyslipidemia, cardiomyopathy, prior stroke or transient ischemic attack, atrial fibrillation and flutter, history of tobacco user, chronic kidney disease, obstructive sleep apnea, migraine, systemic lupus erythematosus, conduction disorders, atherosclerosis, aortic aneurysm and dissection |

## Table A.3. Baseline Characteristics, Demographics, and Comorbidity Profiles of PWE with and without AMI

|  | All hospitalizations  N = 8,456,098 | Acute Myocardial Infarction | | *P* value |
| --- | --- | --- | --- | --- |
|  |  | Absent  N = 8,274,272 | Present  N = 181,826 |  |
| Age, Median (IQR) | 57.00 (27.00) | 57.00 (27.00) | 67.00 (21.00) | <.001 |
| Age group (%) |  |  |  | <.001 |
| 18~44 | 2147921(25.40) | 2137749(25.84) | 10172(5.59) |  |
| 45~64 | 3257613(38.52) | 3190927(38.56) | 66686(36.68) |  |
| 65~74 | 1399264(16.55) | 1354645(16.37) | 44619(24.54) |  |
| ≥75 | 1651301(19.53) | 1590951(19.23) | 60350(33.19) |  |
| Sex (%) |  |  |  | <.001 |
| Male | 3980252(47.07) | 3883882(46.94) | 96370(53.00) |  |
| Female | 4475846(52.93) | 4390390(53.06) | 85456(47.00) |  |
| Race (%) |  |  |  | <.001 |
| White | 5670894(67.06) | 5542859(66.99) | 128034(70.42) |  |
| Black | 1715046(20.28) | 1682424(20.33) | 32622(17.94) |  |
| Hispanic | 696596(8.24) | 683888(8.27) | 12708(6.99) |  |
| Other | 373563(4.42) | 365100(4.41) | 8463(4.65) |  |
| Control/ownership of hospital (%) |  |  |  | <.001 |
| Government nonfederal | 994364(11.76) | 975275(11.79) | 19089(10.50) |  |
| Private not for profit | 6179852(73.08) | 6045956(73.07) | 133896(73.64) |  |
| Private investor owned | 1281882(15.16) | 1253040(15.14) | 28842(15.86) |  |
| Hospital Size (%) |  |  |  | <.001 |
| Small | 1168123(13.81) | 1145250(13.84) | 22873(12.58) |  |
| Medium | 2232315(26.40) | 2183776(26.39) | 48539(26.70) |  |
| Large | 5055660(59.79) | 4945246(59.77) | 110415(60.73) |  |
| Teaching status of hospital (%) |  |  |  | <.001 |
| Nonteaching | 3595026(42.51) | 3519368(42.53) | 75659(41.61) |  |
| Teaching | 4861072(57.49) | 4754904(57.47) | 106168(58.39) |  |
| Location status of hospital (%) |  |  |  | <.001 |
| Rural | 812721(9.61) | 796696(9.63) | 16025(8.81) |  |
| Urban | 7643377(90.39) | 7477575(90.37) | 165802(91.19) |  |
| Region of hospital (%) |  |  |  | <.001 |
| Northeast | 1790264(21.17) | 1754186(21.20) | 36078(19.84) |  |
| Midwest | 1886029(22.30) | 1844478(22.29) | 41551(22.85) |  |
| South | 3341704(39.52) | 3267881(39.49) | 73822(40.60) |  |
| West | 1438101(17.01) | 1407726(17.01) | 30375(16.71) |  |
| Admission day is on a weekend (%) | 1937339(22.91) | 1888828(22.83) | 48510(26.68) | <.001 |
| Season (%) |  |  |  | <.001 |
| Winter | 2347841(27.77) | 2296594(27.76) | 51247(28.18) |  |
| Spring | 2048952(24.23) | 2004513(24.23) | 44439(24.44) |  |
| Summer | 2032274(24.03) | 1990140(24.05) | 42135(23.17) |  |
| Autumn | 2027031(23.97) | 1983026(23.97) | 44005(24.20) |  |
| Primary expected payer (%) |  |  |  | <.001 |
| Medicare | 4694188(55.51) | 4568627(55.21) | 125561(69.06) |  |
| Medicaid | 1818044(21.50) | 1795246(21.70) | 22798(12.54) |  |
| Private insurance | 1374972(16.26) | 1351162(16.33) | 23810(13.09) |  |
| Self-pay | 313084(3.70) | 307992(3.72) | 5091(2.80) |  |
| Other | 255810(3.03) | 251244(3.04) | 4566(2.51) |  |
| Median household income percentile (%) |  |  |  | <.001 |
| <25% | 3122516(36.93) | 3054318(36.91) | 68198(37.51) |  |
| 25%~49% | 2108762(24.94) | 2062328(24.92) | 46434(25.54) |  |
| 50%~74% | 1799464(21.28) | 1761159(21.28) | 38304(21.07) |  |
| ≥75% | 1425356(16.86) | 1396466(16.88) | 28890(15.89) |  |
| Elective admission (%) | 1163572(13.76) | 1152207(13.93) | 11365(6.25) | <.001 |
| Acquired immune deficiency syndrome (%) | 48766(0.58) | 48062(0.58) | 703(0.39) | <.001 |
| Alcohol abuse (%) | 692851(8.19) | 681256(8.23) | 11595(6.38) | <.001 |
| Deficiency anemias (%) | 1853643(21.92) | 1802007(21.78) | 51636(28.40) | <.001 |
| Rheumatoid arthritis/collagen vascular diseases (%) | 261660(3.09) | 256295(3.10) | 5365(2.95) | <.001 |
| Chronic blood loss anemia (%) | 102673(1.21) | 100409(1.21) | 2264(1.25) | .2193 |
| Chronic pulmonary disease (%) | 1934439(22.88) | 1883143(22.76) | 51296(28.21) | <.001 |
| Coagulopathy (%) | 573233(6.78) | 554238(6.70) | 18995(10.45) | <.001 |
| Depression (%) | 1417930(16.77) | 1392140(16.82) | 25790(14.18) | <.001 |
| Diabetes uncomplicated (%) | 1487582(17.59) | 1443299(17.44) | 44283(24.35) | <.001 |
| Diabetes with chronic complications (%) | 567824(6.71) | 545638(6.59) | 22186(12.20) | <.001 |
| Drug abuse (%) | 641926(7.59) | 632185(7.64) | 9741(5.36) | <.001 |
| Hypertension (%) | 4479992(52.98) | 4355999(52.65) | 123992(68.19) | <.001 |
| Hypothyroidism (%) | 1215268(14.37) | 1188795(14.37) | 26473(14.56) | 0.0207 |
| Liver disease (%) | 370148(4.38) | 363766(4.40) | 6382(3.51) | <.001 |
| Lymphoma (%) | 53322(0.63) | 52106(0.63) | 1216(0.67) | 0.0378 |
| Metastatic cancer (%) | 155921(1.84) | 152876(1.85) | 3045(1.67) | <.001 |
| Obesity (%) | 861644(10.19) | 840777(10.16) | 20867(11.48) | <.001 |
| Paralysis (%) | 1062092(12.56) | 1039759(12.57) | 22334(12.28) | <.001 |
| Peripheral vascular disorders (%) | 455474(5.39) | 432011(5.22) | 23463(12.90) | <.001 |
| Psychoses (%) | 1003962(11.87) | 989532(11.96) | 14430(7.94) | <.001 |
| Solid tumor without metastasis (%) | 172741(2.04) | 169030(2.04) | 3711(2.04) | 0.9546 |
| Peptic ulcer disease excluding bleeding (%) | 22179(0.26) | 21514(0.26) | 665(0.37) | <.001 |
| Valvular disease (%) | 316278(3.74) | 306473(3.70) | 9804(5.39) | <.001 |
| Weight loss (%) | 600391(7.10) | 583520(7.05) | 16871(9.28) | <.001 |
| Dyslipidemia (%) | 2299539(27.19) | 2215609(26.78) | 83930(46.16) | <.001 |
| Refectory epilepsy (%) | 215460(2.55) | 213783(2.58) | 1678(0.92) | <.001 |
| Cardiomyopathy (%) | 233603(2.76) | 217402(2.63) | 16201(8.91) | <.001 |
| Prior stroke or transient ischemic attack (%) | 897569(10.61) | 872960(10.55) | 24609(13.53) | <.001 |
| Atrial fibrillation and flutter (%) | 932594(11.03) | 893448(10.80) | 39146(21.53) | <.001 |
| History of tobacco user (%) | 2119653(25.07) | 2069625(25.01) | 50028(27.51) | <.001 |
| Chronic kidney disease (%) | 1228594(14.53) | 1180776(14.27) | 47818(26.30) | <.001 |
| Obstructive sleep apnea (%) | 468498(5.54) | 456957(5.52) | 11541(6.35) | <.001 |
| Migraine (%) | 284274(3.36) | 281956(3.41) | 2319(1.28) | <.001 |
| Systemic lupus erythematosus (%) | 114879(1.36) | 113208(1.37) | 1671(0.92) | <.001 |
| Conduction disorders (%) | 135451(1.60) | 126219(1.53) | 9232(5.08) | <.001 |
| Atherosclerosis (%) | 1550144(18.33) | 1443227(17.44) | 106917(58.80) | <.001 |
| Aortic aneurysm and dissection (%) | 57210(0.68) | 54614(0.66) | 2596(1.43) | <.001 |
| Causing the weight of observation may not be integer, the count of each group may less or more than the total of all group after rounding off. | | | | |

The comparison between the PWE with AMI group and the PWE without AMI group was based on univariate analysis. Age at admission was a continuous variable that did not follow a normal distribution (as assessed by the Kolmogorov-Smirnov test); thus, the Wilcoxon rank-sum test was used for comparison. All other variables were categorical and were compared using the Chi-square test.

AMI, acute myocardial infarction; PWE, patients with epilepsy; IQR, interquartile range.

## Table A.4. Temporal Trends of AMI Prevalence by PWE Subgroups

| Variables | 2008 | 2009 | 2010 | 2011 | 2012 | 2013 | 2014 | 2015 | 2016 | 2017 | *Z* Value | *P_trend_* |
| --- | --- | --- | --- | --- | --- | --- | --- | --- | --- | --- | --- | --- |
| Sex |  |  |  |  |  |  |  |  |  |  |  |  |
| Male | 2.18 | 2.33 | 2.17 | 2.23 | 2.46 | 2.52 | 2.46 | 2.42 | 2.49 | 2.81 | -19.84 | <.0001 |
| Female | 1.68 | 1.86 | 1.80 | 1.79 | 1.86 | 1.96 | 1.83 | 1.88 | 2.07 | 2.27 | -20.01 | <.0001 |
| Age |  |  |  |  |  |  |  |  |  |  |  |  |
| 18~44 | 0.39 | 0.44 | 0.43 | 0.36 | 0.50 | 0.49 | 0.45 | 0.50 | 0.51 | 0.63 | -12.22 | <.0001 |
| 45~64 | 1.79 | 2.04 | 1.81 | 1.84 | 1.99 | 2.07 | 2.17 | 2.01 | 2.18 | 2.47 | -20.44 | <.0001 |
| 65~74 | 2.63 | 3.04 | 3.00 | 2.89 | 3.23 | 3.36 | 3.15 | 3.19 | 3.44 | 3.59 | -15.23 | <.0001 |
| 75 | 3.53 | 3.57 | 3.49 | 3.54 | 3.63 | 3.82 | 3.35 | 3.60 | 3.77 | 4.18 | -9.17 | <.0001 |
| Race |  |  |  |  |  |  |  |  |  |  |  |  |
| White | 2.00 | 2.18 | 2.11 | 2.15 | 2.25 | 2.36 | 2.19 | 2.22 | 2.39 | 2.64 | -22.48 | <.0001 |
| Black | 1.55 | 1.81 | 1.62 | 1.68 | 1.93 | 1.90 | 1.97 | 2.05 | 1.99 | 2.32 | -18.40 | <.0001 |
| Hispanic | 1.56 | 1.78 | 1.57 | 1.66 | 1.80 | 1.85 | 2.03 | 1.72 | 1.87 | 2.19 | -8.96 | <.0001 |
| Other | 2.43 | 2.15 | 2.38 | 1.79 | 2.15 | 2.47 | 2.10 | 2.04 | 2.61 | 2.52 | -3.56 | 0.0004 |
| Insurance type |  |  |  |  |  |  |  |  |  |  |  |  |
| Medicare | 2.37 | 2.57 | 2.52 | 2.49 | 2.68 | 2.75 | 2.60 | 2.65 | 2.83 | 3.13 | -22.70 | <.0001 |
| Medicaid | 1.05 | 1.11 | 0.99 | 1.11 | 1.23 | 1.35 | 1.30 | 1.25 | 1.49 | 1.47 | -18.20 | <.0001 |
| Private insurance | 1.58 | 1.88 | 1.67 | 1.55 | 1.67 | 1.70 | 1.67 | 1.73 | 1.74 | 2.10 | -7.02 | <.0001 |
| Self-pay | 1.32 | 1.62 | 1.41 | 1.44 | 1.44 | 1.82 | 2.12 | 1.50 | 1.65 | 1.97 | -6.76 | <.0001 |
| Other | 1.95 | 1.82 | 1.40 | 1.61 | 1.69 | 1.75 | 1.80 | 2.00 | 1.57 | 2.34 | -3.70 | 0.0002 |
| Hospital region |  |  |  |  |  |  |  |  |  |  |  |  |
| Northeast | 1.90 | 1.93 | 1.72 | 1.92 | 2.02 | 2.10 | 2.05 | 1.99 | 2.14 | 2.34 | -12.21 | <.0001 |
| Midwest | 1.81 | 1.91 | 2.13 | 2.02 | 2.10 | 2.22 | 2.21 | 2.32 | 2.45 | 2.65 | -20.89 | <.0001 |
| South | 1.96 | 2.26 | 1.99 | 1.93 | 2.26 | 2.30 | 2.20 | 2.18 | 2.27 | 2.63 | -17.59 | <.0001 |
| West | 1.94 | 2.11 | 2.06 | 2.20 | 2.06 | 2.21 | 1.93 | 1.96 | 2.20 | 2.36 | -4.75 | <.0001 |
| Median household income percentile |  |  |  |  |  |  |  |  |  |  |  |  |
| <25% | 1.79 | 2.07 | 1.96 | 1.93 | 2.20 | 2.30 | 2.19 | 2.22 | 2.34 | 2.66 | -24.74 | <.0001 |
| 25%~49% | 1.91 | 2.04 | 2.02 | 2.07 | 2.16 | 2.27 | 2.20 | 2.20 | 2.42 | 2.57 | -17.38 | <.0001 |
| 50%~74% | 1.98 | 2.07 | 2.08 | 2.01 | 2.05 | 2.19 | 2.11 | 1.98 | 2.16 | 2.57 | -9.77 | <.0001 |
| ≥75% | 2.09 | 2.20 | 1.80 | 1.99 | 2.09 | 2.03 | 1.89 | 2.05 | 2.03 | 2.13 | -0.53 | 0.5962 |

Values are %

The significance of trends over time was assessed using the Cochran-Armitage trend test.

AMI, acute myocardial infarction; PWE, patients with epilepsy.

## Table A.5. Outcomes of PWE with and without AMI

| Items | All hospitalizations  N = 8,456,098 | Acute Myocardial Infarction | | *P* value |
| --- | --- | --- | --- | --- |
|  |  | Absent  N = 8,274,272 | Present  N = 181,826 |  |
| Adverse outcomes (%) |  |  |  |  |
| In-patient death | 230864(2.73) | 206598(2.50) | 24266(13.35) | <.001 |
| Acute heart failure | 268370(3.17) | 238446(2.88) | 29924(16.46) | <.001 |
| Acute respiratory failure | 976013(11.54) | 917310(11.09) | 58703(32.29) | <.001 |
| Acute renal failure | 1032345(12.21) | 980364(11.85) | 51981(28.59) | <.001 |
| Neurological failure | 1044463(12.35) | 1001289(12.10) | 43174(23.74) | <.001 |
| Cardiogenic shock | 25298(0.30) | 15167(0.18) | 10130(5.57) | <.001 |
| Length of stay, days, median(IQR) | 4.00(4.00) | 4.00(4.00) | 6.00(7.00) | <.001 |
| Total cost, USD, median(IQR) | 31258.86(41741.57) | 30929.86(40710.14) | 64872.25(87849.54) | <.001 |
| Patient disposition(%) |  |  |  | <.001 |
| Routine | 4356766(51.52) | 4293396(51.89) | 63370(34.85) |  |
| Transfer to Short-term Hospital | 33363(0.39) | 31766(0.38) | 1597(0.88) |  |
| Transfer to skilled nursing facility | 2509189(29.67) | 2446087(29.56) | 63102(34.70) |  |
| Or Home Health Care | 1204404(14.24) | 1176819(14.22) | 27584(15.17) |  |
| Against Medical Advice | 116530(1.38) | 114809(1.39) | 1721(0.95) |  |
| In-hospital death | 230864(2.73) | 206598(2.50) | 24266(13.35) |  |
| Discharge alive destination unknown | 4982(0.06) | 4797(0.06) | 185(0.10) |  |
| Causing the weight of observation may not be integer, the count of each group may less or more than the total of all group after rounding off. | | | | |

The comparison between the PWE with AMI group and the PWE without AMI group was based on univariate analysis. Length of stay and total cost were continuous variables which did not follow a normal distribution (as assessed by the Kolmogorov-Smirnov test); thus, the Wilcoxon rank-sum test was used for comparison. All other variables were categorical and were assessed using the Chi-square test.

AMI, acute myocardial infarction; PWE, patients with epilepsy; USD, US dollars; IQR, interquartile range.

## Table A.6. Associations of concomitant AMI with Adverse Clinical Outcomes in PWE

| Adverse Clinical Outcome | Model 1  (Unadjusted Model) | | Model 2  (Main Analysis) | | Model 3  (Sensitive Analysis) | |
| --- | --- | --- | --- | --- | --- | --- |
|  | OR (95% CI) | *P* Value | OR (95% CI) | *P* Value | OR (95% CI) | *P* Value |
| In-patient death | 6.02(5.93-6.10) | <.0001 | 4.58(4.51-4.65) | <.0001 | 4.56(4.49-4.64) | <.0001 |
| Acute Heart Failure | 6.64(6.55-6.73) | <.0001 | 3.51(3.46-3.56) | <.0001 | 3.49(3.43-3.54) | <.0001 |
| Acute Respiratory Failure | 3.82(3.79-3.86) | <.0001 | 3.44(3.40-3.47) | <.0001 | 3.39(3.35-3.43) | <.0001 |
| Acute Renal Failure | 2.98(2.95-3.01) | <.0001 | 2.19(2.16-2.21) | <.0001 | 2.17(2.14-2.19) | <.0001 |
| Neurological Failure | 2.26(2.24-2.29) | <.0001 | 1.91(1.89-1.93) | <.0001 | 1.88(1.86-1.90) | <.0001 |
| Cardiogenic Shock | 32.13(31.32-32.97) | <.0001 | 17.74(17.24-18.25) | <.0001 | 17.38(16.86-17.91) | <.0001 |

Model 1: ORs and P value were obtained from univariate logistic regression models with each adverse outcome as the dependent variable and AMI as the independent variable. The analysis accounted for the survey design and was performed on the original dataset without imputation.

Model 2: ORs and P value were obtained from multivariate logistic regression models with each adverse outcome as the dependent variable and AMI as the independent variable, adjusted for patient-level factors (sex, age, race, insurance type, income level, discharge year and season), hospital-level factors (hospital ownership, hospital region, hospital size, hospital location, teaching status), and comorbidity related factors. The analysis accounted for the survey design and was based on the imputed dataset (missing values in category variables were imputed by the dominant category, and missing values in continuous variables were imputed by median value.).

Model 3: Same as Model 2, with the only difference being that Model 3 is based on the original dataset without imputation.

AMI, acute myocardial infarction; PWE, patients with epilepsy; OR, odds ratio;

## Table A.7. Associations of Clinical Factors with concomitant AMI in PWE

| Variables | Model1  (Unadjusted Model) | | Model 2  (Main Analysis) | | Model 3  (Sensitive Analysis) | |
| --- | --- | --- | --- | --- | --- | --- |
|  | OR (95% CI) | *P* Value | OR (95% CI) | *P* value | OR (95% CI) | *P* value |
| Age group | | | | | | |
| 18~44 | (Reference) |  | (Reference) |  | (Reference) |  |
| 45~64 | 4.39(4.30-4.48) | <.0001 | 2.82(2.76-2.88) | <.0001 | 2.78(2.71-2.84) | <.0001 |
| 65~74 | 6.92(6.77-7.07) | <.0001 | 3.34(3.26-3.42) | <.0001 | 3.29(3.20-3.37) | <.0001 |
| ≥75 | 7.97(7.80-8.14) | <.0001 | 3.54(3.45-3.62) | <.0001 | 3.45(3.36-3.54) | <.0001 |
| Female | 0.78(0.78-0.79) | <.0001 | 0.96(0.95-0.97) | <.0001 | 0.96(0.95-0.97) | <.0001 |
| Race | | | | | | |
| White | (Reference) |  | (Reference) |  | (Reference) |  |
| Black | 0.84(0.83-0.85) | <.0001 | 0.78(0.77-0.79) | <.0001 | 0.78(0.77-0.79) | <.0001 |
| Hispanic | 0.80(0.79-0.82) | <.0001 | 0.86(0.84-0.87) | <.0001 | 0.86(0.84-0.88) | <.0001 |
| Other | 1.00(0.98-1.03) | 0.7611 | 1.03(1.00-1.05) | 0.0184 | 1.03(1.00-1.05) | 0.0326 |
| Primary expected pay | | | | | | |
| Medicare | (Reference) |  | (Reference) |  | (Reference) |  |
| Medicaid | 0.46(0.46-0.47) | <.0001 | 1.00(0.98-1.01) | 0.5557 | 0.99(0.97-1.01) | 0.1811 |
| Private ins | 0.64(0.63-0.65) | <.0001 | 1.18(1.16-1.20) | <.0001 | 1.17(1.15-1.19) | <.0001 |
| Self-pay | 0.60(0.58-0.62) | <.0001 | 1.41(1.37-1.45) | <.0001 | 1.41(1.36-1.45) | <.0001 |
| Other | 0.66(0.64-0.68) | <.0001 | 1.17(1.13-1.20) | <.0001 | 1.16(1.12-1.20) | <.0001 |
| Median household income percentile | | | | | | |
| <25% | (Reference) |  | (Reference) |  | (Reference) |  |
| 25%~49% | 1.01(1.00-1.02) | 0.1711 | 0.96(0.95-0.97) | <.0001 | 0.95(0.93-0.96) | <.0001 |
| <50%~74% | 0.97(0.96-0.99) | <.0001 | 0.90(0.89-0.91) | <.0001 | 0.89(0.87-0.90) | <.0001 |
| ≥75% | 0.93(0.91-0.94) | <.0001 | 0.83(0.82-0.84) | <.0001 | 0.81(0.80-0.83) | <.0001 |
| Control/ownership of hospital | | | | | | |
| Government non-federal | (Reference) |  | (Reference) |  | (Reference) |  |
| Private not for profit | 1.13(1.11-1.15) | <.0001 | 0.97(0.95-0.98) | <.0001 | 0.95(0.93-0.97) | <.0001 |
| Private investor owned | 1.18(1.15-1.20) | <.0001 | 1.02(1.00-1.04) | 0.0155 | 1.01(0.99-1.03) | 0.3770 |
| Hospital Size | | | | | | |
| Small | (Reference) |  | (Reference) |  | (Reference) |  |
| Medium | 1.11(1.10-1.13) | <.0001 | 1.10(1.09-1.12) | <.0001 | 1.09(1.08-1.11) | <.0001 |
| Large | 1.12(1.10-1.13) | <.0001 | 1.17(1.15-1.19) | <.0001 | 1.15(1.13-1.17) | <.0001 |
| Teaching hospital | 1.04(1.03-1.05) | <.0001 | 1.15(1.14-1.16) | <.0001 | 1.15(1.14-1.16) | <.0001 |
| Urban hospital | 1.10(1.08-1.12) | <.0001 | 1.10(1.08-1.12) | <.0001 | 1.11(1.09-1.14) | <.0001 |
| Admission day is on weekend | 1.23(1.22-1.24) | <.0001 | 1.21(1.20-1.23) | <.0001 | 1.22(1.20-1.23) | <.0001 |
| Season | | | | | | |
| Winter | (Reference) |  | (Reference) |  | (Reference) |  |
| Spring | 0.99(0.98-1.01) | 0.3197 | 1.01(0.99-1.02) | 0.2924 | 0.98(0.97-1.00) | 0.0196 |
| Summer | 0.95(0.94-0.96) | <.0001 | 0.97(0.96-0.98) | <.0001 | 0.95(0.94-0.96) | <.0001 |
| Autumn | 0.99(0.98-1.01) | 0.3976 | 1.02(1.00-1.03) | 0.0097 | 0.99(0.98-1.01) | 0.2337 |
| Region of hospital | | | | | | |
| Northeast | (Reference) |  | (Reference) |  | (Reference) |  |
| Midwest | 1.10(1.08-1.11) | <.0001 | 1.00(0.99-1.02) | 0.7273 | 1.01(0.99-1.02) | 0.3008 |
| South | 1.10(1.08-1.11) | <.0001 | 1.02(1.00-1.03) | 0.0301 | 1.02(1.00-1.03) | 0.0216 |
| West | 1.05(1.03-1.07) | <.0001 | 1.03(1.01-1.05) | 0.0002 | 1.03(1.02-1.05) | 0.0001 |
| Dyslipidaemia | 2.34(2.32-2.37) | <.0001 | 1.26(1.24-1.27) | <.0001 | 1.25(1.23-1.26) | <.0001 |
| Refectory epilepsy | 0.35(0.34-0.37) | <.0001 | 0.68(0.64-0.71) | <.0001 | 0.70(0.67-0.74) | <.0001 |
| Hypertension | 1.93(1.91-1.95) | <.0001 |  |  |  |  |
| Diabetes uncomplicated | 1.52(1.51-1.54) | <.0001 | 1.08(1.06-1.09) | <.0001 | 1.08(1.06-1.09) | <.0001 |
| Diabetes with chronic complications | 1.97(1.94-2.00) | <.0001 | 1.15(1.13-1.16) | <.0001 | 1.14(1.12-1.16) | <.0001 |
| Obesity | 1.15(1.13-1.16) | <.0001 | 1.09(1.07-1.11) | <.0001 | 1.09(1.08-1.11) | <.0001 |
| History of tobacco user | 1.14(1.13-1.15) | . | 1.20(1.18-1.21) | <.0001 | 1.20(1.18-1.21) | <.0001 |
| Drug abuse | 0.68(0.67-0.70) | <.0001 | 1.18(1.16-1.21) | <.0001 | 1.20(1.17-1.23) | <.0001 |
| Alcohol abuse | 0.76(0.75-0.77) | <.0001 | 0.91(0.89-0.93) | <.0001 | 0.92(0.90-0.94) | <.0001 |
| Weight loss | 1.35(1.33-1.37) | <.0001 | 1.20(1.18-1.22) | <.0001 | 1.20(1.18-1.22) | <.0001 |
| Prior stroke or transient ischemic attack | 1.33(1.31-1.35) | <.0001 | 0.82(0.81-0.84) | <.0001 | 0.83(0.82-0.84) | <.0001 |
| Atrial fibrillation and flutter | 2.27(2.24-2.29) | <.0001 | 1.12(1.11-1.13) | <.0001 | 1.12(1.11-1.14) | <.0001 |
| Valvular disease | 1.48(1.45-1.51) | <.0001 | 0.82(0.80-0.84) | <.0001 | 0.82(0.81-0.84) | <.0001 |
| Cardiomyopathy | 3.63(3.57-3.69) | <.0001 | 2.11(2.08-2.15) | <.0001 | 2.09(2.05-2.13) | <.0001 |
| Conduction disorders | 3.81(3.74-3.88) | <.0001 | 2.21(2.17-2.26) | <.0001 | 2.24(2.19-2.28) | <.0001 |
| Atherosclerosis | 6.76(6.69-6.82) | <.0001 | 4.44(4.40-4.49) | <.0001 | 4.42(4.37-4.48) | <.0001 |
| Aortic aneurysm and dissection | 2.18(2.10-2.27) | <.0001 | 0.95(0.91-0.99) | 0.0107 | 0.95(0.91-0.99) | 0.0290 |
| Obstructive sleep apnea | 1.16(1.14-1.18) | <.0001 | 0.79(0.78-0.81) | <.0001 | 0.81(0.79-0.82) | <.0001 |
| Chronic pulmonary disease | 1.33(1.32-1.35) | <.0001 |  |  | 0.99(0.97-1.00) | 0.0224 |
| Peptic ulcer disease excluding bleeding | 1.41(1.31-1.52) | <.0001 | 1.23(1.13-1.33) | <.0001 | 1.23(1.13-1.33) | <.0001 |
| Liver disease | 0.79(0.77-0.81) | <.0001 | 0.81(0.79-0.83) | <.0001 | 0.81(0.79-0.83) | <.0001 |
| Paralysis | 0.97(0.96-0.99) | 0.0004 | 0.98(0.96-0.99) | 0.0018 | 0.98(0.96-0.99) | 0.0034 |
| Psychoses | 0.63(0.62-0.65) | <.0001 | 0.75(0.74-0.76) | <.0001 | 0.75(0.73-0.76) | <.0001 |
| Migraine | 0.37(0.35-0.38) | <.0001 | 0.61(0.59-0.64) | <.0001 | 0.60(0.58-0.63) | <.0001 |
| Depression | 0.82(0.81-0.83) | <.0001 | 0.76(0.75-0.78) | <.0001 | 0.76(0.75-0.77) | <.0001 |
| Chronic kidney disease | 2.14(2.12-2.17) | <.0001 | 1.23(1.22-1.25) | <.0001 | 1.23(1.21-1.25) | <.0001 |
| Peripheral vascular disorders | 2.69(2.65-2.73) | <.0001 | 1.09(1.08-1.11) | <.0001 | 1.08(1.06-1.10) | <.0001 |
| Systemic lupus erythematosus | 0.67(0.64-0.70) | <.0001 |  |  |  |  |
| Acquired immune deficiency syndrome | 0.67(0.62-0.72) | <.0001 |  |  |  |  |
| Deficiency anemias | 1.42(1.41-1.44) | <.0001 | 1.08(1.06-1.09) | <.0001 | 1.08(1.06-1.09) | <.0001 |
| Rheumatoid arthritis/collagen vascular diseases | 0.95(0.93-0.98) | 0.0005 | 0.96(0.93-0.99) | 0.0036 | 0.95(0.92-0.98) | 0.0011 |
| Chronic blood loss anemia | 1.03(0.98-1.07) | 0.2136 | 1.10(1.05-1.14) | <.0001 | 1.05(1.01-1.10) | 0.0300 |
| Coagulopathy | 1.62(1.60-1.65) | <.0001 | 1.52(1.49-1.54) | <.0001 | 1.51(1.48-1.54) | <.0001 |
| Hypothyroidism | 1.02(1.00-1.03) | 0.0200 | 0.83(0.81-0.84) | <.0001 | 0.82(0.81-0.83) | <.0001 |
| Lymphoma | 1.06(1.01-1.13) | 0.0325 | 0.85(0.80-0.90) | <.0001 | 0.83(0.78-0.88) | <.0001 |
| Metastatic cancer | 0.90(0.87-0.94) | <.0001 | 0.85(0.82-0.88) | <.0001 | 0.84(0.81-0.87) | <.0001 |
| Solid tumor without metastasis | 1.00(0.97-1.03) | 1.0000 | 0.88(0.85-0.91) | <.0001 | 0.88(0.85-0.92) | <.0001 |

Model 1: ORs and P value were obtained from univariate logistic regression models with AMI as the dependent variable and each clinical factors as the independent variable. The analysis accounted for the survey design and was performed on the original dataset without imputation.

Model 2: ORs and P value were obtained from multivariate logistic regression models with AMI as the dependent variable and each clinical factors as the independent variable, adjusted for other clinical factors, including patient-level factors, hospital-level factors, and comorbidity related factors. The analysis accounted for the survey design and was based on the imputed dataset (missing values in category variables were imputed by the dominant category, and missing values in continuous variables were imputed by median value.).

Model 3: Same as Model 2, with the only difference being that Model 3 is based on the original dataset without imputation.

AMI, acute myocardial infarction; PWE, patients with epilepsy; OR, odds ratio.

## Table A.8. Associations of concomitant STEMI / NSTEMI with Adverse Clinical Outcomes in PWE

| Adverse Clinical Outcome | PWE + STEMI vs PWE only | | PWE+NSTEMI vs PWE only | |
| --- | --- | --- | --- | --- |
|  | OR (95% CI) | P Value | OR (95% CI) | P Value |
| In-patient death | 9.07(8.78-9.37) | <.0001 | 3.94(3.87-4.00) | <.0001 |
| Acute Heart Failure | 4.69(4.51-4.88) | <.0001 | 4.04(3.98-4.10) | <.0001 |
| Acute Respiratory Failure | 3.77(3.66-3.87) | <.0001 | 3.23(3.19-2.27) | <.0001 |
| Acute Renal Failure | 2.21(2.14-2.28) | <.0001 | 2.12(2.09-2.14) | <.0001 |
| Neurological Failure | 2.51(2.44-2.58) | <.0001 | 1.75(1.73-1.77) | <.0001 |
| Cardiogenic Shock | 89.87(86.23-93.66) | <.0001 | 13.54(13.11-13.99) | <.0001 |

Obtained from multivariate logistic regression models with each adverse outcome as the dependent variable and either concomitant STEMI or NSTEMI as the independent variable, adjusted for patient-level factors (sex, age, race, insurance type, income level, discharge year and season), hospital-level factors (hospital ownership, hospital region, hospital size, hospital location, teaching status), and comorbidity related factors. The analysis accounted for the survey design and was based on the imputed dataset (missing values in category variables were imputed by the dominant category, and missing values in continuous variables were imputed by median value.).

STEMI, ST-segment elevation myocardial infarction; NSTEMI, non-ST-segment elevation myocardial infarction; PWE, patients with epilepsy.


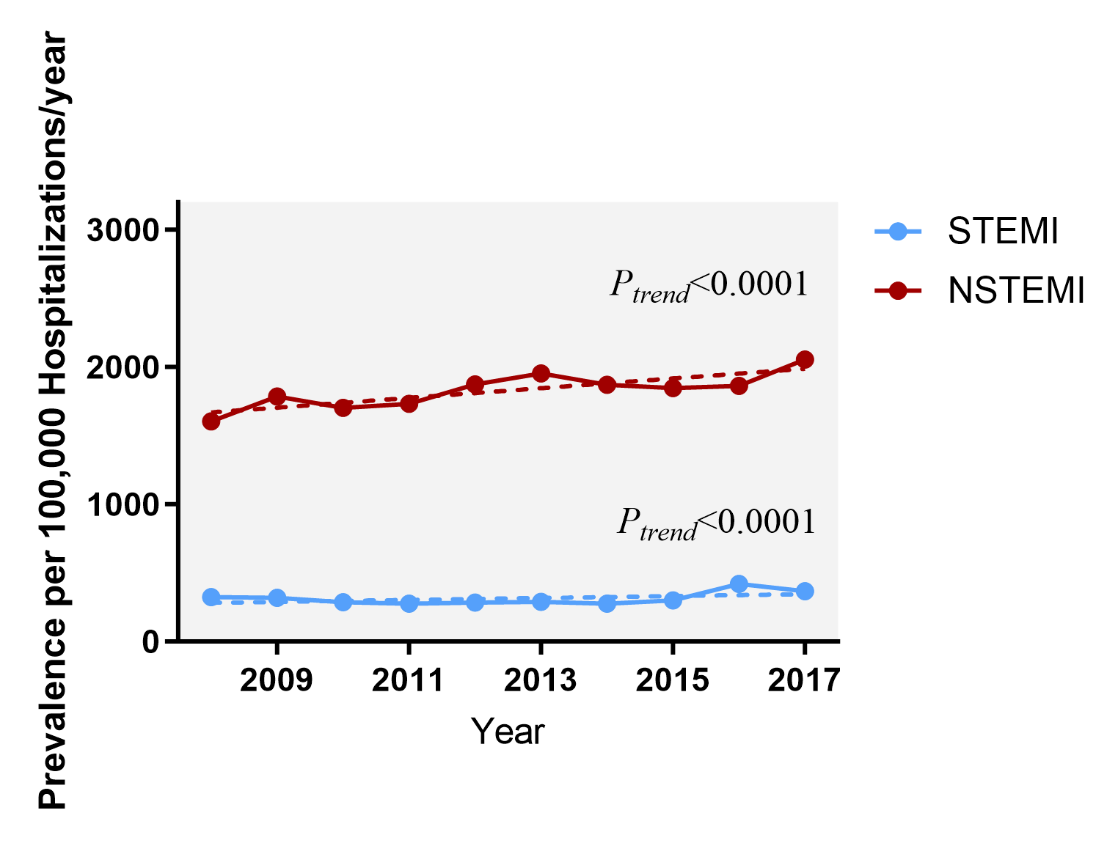


## Figure A.1 Temporal Trends of STEMI/NSTEMI Prevalence in PWE

The significance of trends over time was assessed using the Cochran-Armitage trend test.

STEMI, ST-segment elevation myocardial infarction; NSTEMI, non-ST-segment elevation myocardial infarction; PWE, patients with epilepsy.
